# Length and density of filiform tongue papillae: differences between tick-susceptible and resistant cattle may affect tick loads

**DOI:** 10.1186/s13071-015-1196-4

**Published:** 2015-11-16

**Authors:** Cecília José Veríssimo, Selma Marques D’Agostino, Fernanda Ferreira Pessoa, Luciandra Macedo de Toledo, Isabel Kinney Ferreira de Miranda Santos

**Affiliations:** São Paulo Animal Science Institute, Rua Heitor Penteado 56, Nova Odessa, São Paulo, SP 13460-000 Brazil; Ribeirão Preto School of Medicine, University of São Paulo, Ribeirao Preto, São Paulo, Brazil

**Keywords:** Cattle, Grooming behavior, Resistance, Ticks, Zebu, Taurine

## Abstract

**Background:**

Indicine breeds of bovines are highly resistant and taurine breeds are susceptible to the cattle tick, *Rhipicephalus microplus*, a species which causes great damage to livestock. Animals use their tongues for self-grooming, an important behavior for ridding themselves of ectoparasites. However, the role of tongue morphology, notably the filiform papillae, in this process is not known.

**Findings:**

This study compared features of the filiform papillae of tongues in eight Nelores (indicine breed) and eight Holsteins and two Brown Swiss (taurine breeds) and verified how they associate with tick loads. Biopsies were taken from identical positions of tongues and measured by scanning electron microscopy*.* One-way analysis of variance detected significant differences between morphological features of tongues from indicine and taurine breeds: Nelores had longer papillae (mean of 2.3 mm ± 0.029 SD; *P* < 0.001), and more papillae per cm^2^ (mean of 25.2 papillae ± 1.92 SD; *P* < 0.05) than European bovines (means of, respectively, 1.8 mm ± 0.027 SD and 20.9 ± 0.74 SD papillae per cm^2^). After infestations with equal numbers of larvae, loads of adult ticks were inversely correlated with length of papillae and directly correlated with distances between the apices of papillae (*P* = 0.014; *r* = −0.566 and *P* = 0.018; *r* = 0.567, Pearson product momentum correlation, respectively).

**Conclusions:**

Spacing between papillae is smaller in Nelores, thus their tongues may be rougher and, consequently, more effective in removing tick larvae during self-grooming, explaining the greater resistance to ticks among Zebu breeds of cattle.

## Findings

### Background and hypothesis

The importance of self-grooming for reducing tick infestations in cattle is well documented and is one of the most important defense mechanisms against this parasite [1–6]. The tongue is the main tool that ruminants and other animals use for grooming [7–11].

The cattle tick, *Rhipicephalus microplus*, is one of the most harmful among the ectoparasites that affect production animals. It gives rise to great losses of livestock production in Brazil [12] and in other countries where this parasite is present [13]. It is well known that indicine breeds are resistant and taurine breeds are susceptible to this tick [14]. However, there is a lack of knowledge about the role of grooming in reducing tick infestations. In particular, it remains unknown whether there are any differences in the morphology of these breeds’ tongues that might affect the efficacy of the grooming process. The bovine tongue has four types of papillae: filiform, fungiform, foliate and circumvallate forms. The filiform papillae are located mainly in the anterior third of the tongue and are also used for grooming [15].

The hypothesis of the present study is that morphological features of tongues differ between tick-resistant and tick-susceptible breeds of bovines in such a manner that grooming and tick removal is more efficient in resistant breeds. The present study’s aims are to describe and compare the filiform papilla from an indicine breed (Nelore) and two taurine breeds (Holstein and Brown Swiss) of cattle and to correlate the possible differences in tongue morphology observed in each breed with the tick loads they present.

### Methods

#### Ethical approval

The procedures employed in this study were approved by the Ethics Committee for Animal Experimentation of the Institute of Animal Science (protocol number 144/2011).

A total of 18 steers (approximately two years old) were studied. They belonged to one indicine breed (Nelore; *N* = 8) and to two European breeds (Holstein; *N* = 8; Brown Swiss; *N* = 2). Using an 8 mm diameter punch, a biopsy was taken from the tongues in their central anterior third, at a distance of 3 cm from the tips. The animals were managed together at the Institute of Animal Science (Nova Odessa, state of São Paulo) and were fed with forage hay from the age of 6 months onwards. The biopsy was performed after the animals had been anaesthetized with 2 % xylazine hydrochloride and 2.0 g of lidocaine hydrochloride. After the tissue had been removed, the local lesions were treated with triamcinolone acetonide ointment (1.0 mg g^−1^) and after sedation ceased all animals were eating and drinking normally.

Immediately after collection, the biopsies were kept in saline and were taken to the laboratory, where they were cleaned to remove impurities and dipped in Karnovsky solution (2.5 % glutaraldehyde; formaldehyde; 2.5 % cacodylate in 0.05 M sodium buffer, pH 7.2; 0.001 M CaCl_2_). The samples were then stored in a refrigerator until the assessment procedure was performed using a scanning electron microscope (Zeiss LEO VP-435, Cambridge) at the Electron Microscopy Support Center for Applied Agricultural Research of the Agronomy School of the University of São Paulo, Piracicaba, SP, Brazil).

The samples were dehydrated in solutions of increasing concentrations of acetone (30, 50, 70, 90 and 100 %). They were left in each of these solutions for approximately 15 min (30 and 50 %), 30 min (70 %) and 10 min (90 and 100 %), and were then washed three times (10 min each). After this process, the samples were moistened in a solution of 100 % acetone, critical-point dried (Baltec CPD 030 apparatus), glued onto stubs, metallized with gold (Baltec SCD 050 sputter apparatus) and kept in a container with silica gel.

Inside the microscope, all the stubs were turned so as to view the papillae in the caudal direction, since these papillae were originally on the dorsal surface of the tongue before being collected. They were then measured in terms of their length and base width and the distance between bases and between tips, with the aid of a tool for measurements between two points that is available in the Zeiss scanning electron microscope software (Zeiss, Cambridge). At least ten measurements were made for each item. We also calculated the number of filiform papillae per cm^2^, based on the number of papillae contained in the biopsy area.

We performed statistical analyses using the SPSS*®* package (version 12.0), using a completely randomized design. One-way analysis of variance was performed to detect significant differences between the breeds (indicine versus taurine), in relation to the means for the filiform papilla dimensions and the number of filiform papillae per cm^2^. Pearson’s product moment was used to ascertain whether there were any significant correlations between different aspects of papillae morphology and tick loads.

Before obtaining the samples, an evaluation of tick loads was carried out by means of artificial infestation of each taurine steer with 10,000 larvae and of each indicine steer with 20,000 larvae. The number of female ticks bigger than 4 mm on the entire body of each animal was counted on three nonconsecutive days (days 20, 22 and 24 post-infestation). One-way analysis of variance was performed to detect any significant differences between the breeds regarding the number of ticks. Significance of associations between tick loads and measurements of tongue feature was tested with the Pearson product momentum correlation.

### Results and discussion

**Fig. 1:**
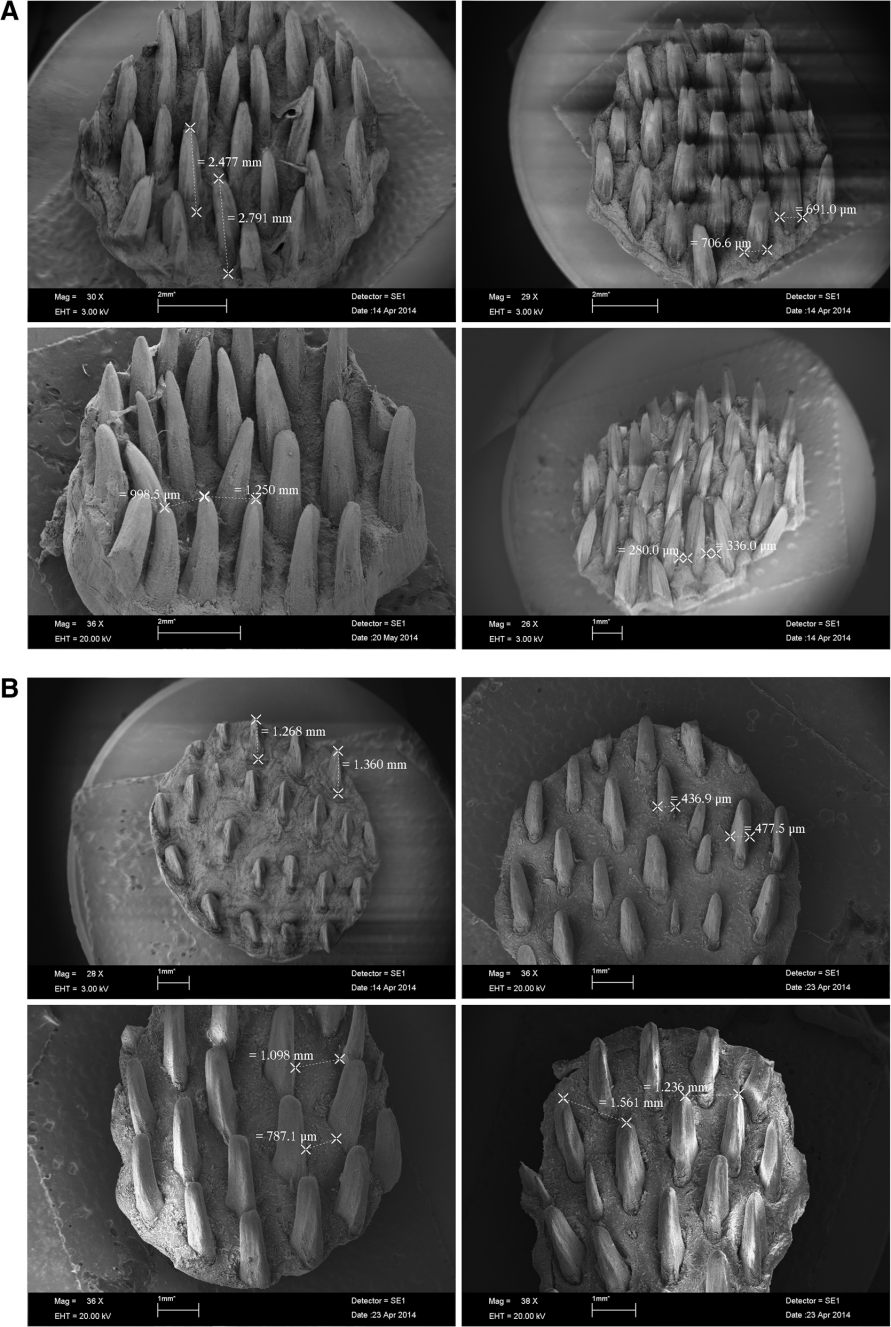
**a** Representative scanning electron micrographs of tongue biopsies from four different cattle of the Nelore indicine breed indicating where the measurements on papillae were made; **b** Representative electron scanning micrographs of tongue biopsies from four different cattle of the two taurine breeds (Holstein and Brown Swiss) indicating where the measurements on papillae were made

Figure 1a and b show the scanning electron micrographs of tongue papillae from four tick-resistant Nelore steers and four tick-susceptible taurine steers, respectively. The measurements and the respective tick counts are presented in Table 1. They show that the tongues of the Nelore (tick-resistant) breed contain significantly longer filiform papillae (mean of 2.3 mm ± 0.029 SD; *P* < 0.001, one-way ANOVA) and significantly more filiform papillae per cm^2^ (mean of 25.2 papillae ± 1.92 SD; *P <* 0.05, one-way ANOVA) than the European breeds of cattle (means of, respectively, 1.8 mm ± 0.027 SD and 20.9 ± 0.74 SD papillae per cm^2^).

**Table 1:** Morphological features of filiform papillae from indicine cattle (Nelore breed) and taurine cattle (Holstein and Brown Swiss breeds). Means (± standard error) and P values for length, base width, distances between bases and between tips (all measurements in mm), number of papillae per cm^2^ and mean number of ticks

Breed	Length	Base width	Distance between bases	Distance between apices	N^o^ of papillae/cm^2^	N^o^ of female ticks ≥ 4 mm
Nelore	2.275 (0.029)	0.625 (0.011)	0.457 (0.014)	0.974 (0.025)	25.22 (1.92)	104.5 (31.22)
Holstein and Swiss Brown	1.789 (0.027)	0.620 (0.009)	0.652 (0.016)	1.326 (0.027)	20.90 (0.74)	515.6 (58.96)
P > F	*<0.001*	0.722	*< 0.001*	*< 0.001*	*< 0.05*	*< 0.001*

Studies in which the sizes of the filiform papillae in tongues from European taurine cattle were measured have found papilla lengths of between 1.5 and 2 mm, and base widths ranging from 0.2 to 0.5 mm [16–19].

Because there were more filiform papillae per unit area in the tongues of the tick-resistant Nelore steers, the distances between the apices of the papillae were significantly smaller (0.974 ± 0.025 mm; *P* < 0.001) than in the tick-susceptible taurine breeds (1.326 ± 0.027 mm). The distances between the bases of the papillae also differed significantly between the indicine and taurine breeds (*P* < 0.001), but the base widths were similar in all the breeds examined in this study.

**Fig. 2:**
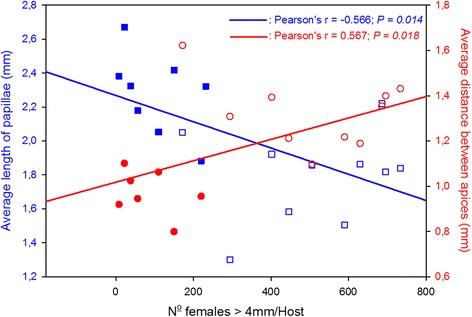
Correlation of tick loads with length of papillae (*blue squares*) and with distances between apices of papillae (*red circles*). Filled symbols represent data from the Nelore tick-resistant breed and open symbols represent data from the two taurine (Holstein and Brown Swiss) tick-susceptible breeds

Interestingly, there was a significant inverse correlation (*P* = 0.014; *r* = −0.566, Pearson product momentum correlation) between tick loads and length of papillae; and there were significant direct correlations (*P* = 0.018; *r* = 0.567, Pearson product momentum correlation) between tick loads and distances between the apices of papillae (Fig. 2) and between tick loads and distances between the bases of the papillae (data not shown). Conversely, no significant correlation was found between tick loads and density of papillae (i.e. number of papillae/cm^2^).

This morphology should give greater roughness to the Nelore tongue and consequently more effectiveness in removing tick larvae during self-grooming. It could be one of the explanations for the greater resistance to ticks seen in Zebu cattle, since self-cleaning is an important cattle defense against *R. microplus* ticks [3–6]. To support this theory, we have found (D’Agostino unpublished data) that Holstein heifers with more filiform papillae per area had fewer ticks than did those with fewer papillae per unit area.

It was interesting to note that at least one Zebu animal (Fig. 1a) presented bipartite tips on many filiform papillae. Shao and colleagues [19] also found serrated and jagged surfaces on the tips of filiform papillae of *Bos taurus* in Tibet. They attributed this to the mechanical function of the papillae, which is to aid in apprehension of food. Their caudal orientation contributes towards leading the food to the esophagus, before swallowing. D’Agostino (unpublished data) found one Holstein heifer with this bipartite tip on some filiform papillae.

### Conclusions

Indicine cattle have more filiform papillae per unit area, and these are longer than those of taurine cattle and the apices are more closely grouped. This should promote greater roughness of the tongue and increase the effectiveness of self-grooming towards removing tick larvae and other parasites from the body, and could be one of the explanations for the greater resistance of indicine cattle breeds to ticks.

## Abbreviations

SEM: Scanning electron microscopy
